# Indigenous Management Practices to Reduce Pests and Pathogens of Cash Crops in Agroforestry Systems

**DOI:** 10.1002/ece3.73094

**Published:** 2026-02-12

**Authors:** Marco Campera, Jake Skull, Lizzie H. Morton, Charlotte A. Grant, Diogo De Almeida Santos, Dylan Wimble, Aislinn Olthoff, Lilli Stenger, Joel Bowden‐Pickstock, I. Made Setiawan, I. Ketut Maliawan, Angel Sangha, Jessica Chavez, Mia Garza, Jenna Sleath‐Probets, Aikaterina Karageorgiadi, Made Dwi Sadnyana, I. Putu Leo Ardhiyanto, Michela Balestri, Andrew K. Jones, Sophie Manson, Muhammad Syirazi, Zefanya Ajiningrat Wibowo, Kuntayuni, Vincent Nijman, Matthew W. Bulbert, Vinni Jain, Desak Ketut Tristiana Sukmadewi

**Affiliations:** ^1^ Sustainable Agroforestry Research Group, School of Biological and Medical Sciences Oxford Brookes University Oxford UK; ^2^ School of Law and Social Sciences Oxford Brookes University Oxford UK; ^3^ Bumi Lestari Conservana Gianyar Indonesia; ^4^ EcoVerde Global Consultancy Sacramento CA USA; ^5^ Taman Nusantara Lestari Jembrana Indonesia; ^6^ College of Agriculture and Life Sciences Cornell University Ithaca NY USA; ^7^ Warmadewa University Denpasar Indonesia; ^8^ Agrotechnology Study Program, Faculty of Agriculture Universitas Warmadewa Denpasar Indonesia

**Keywords:** community forest, fungal disease, indigenous knowledge, Indonesia, local ecological knowledge, polyculture

## Abstract

Indigenous farming practices (e.g., traditional agroforestry) can sustain high levels of biodiversity and ecosystem services (e.g., natural pest and pathogen control) and can be sustainable in the long term, especially if integrated with innovation and technology. We investigate the factors influencing pests and pathogen impact on agroforestry practices in Indigenous communities in Bali, Indonesia. We collected data via 100 plots with different compositions of crops in both community forests (where the use of agrochemicals is precluded by social rules) and polyculture (with no limitation on the use of agrochemicals). For each plot, we obtained a score of infestation for four cash crops (banana, coffee, cacao, and vanilla) by calculating the proportion of plants affected and giving a percentage score of infestation. Analysis was performed via generalised additive models with crop richness, canopy cover, and agroforestry type as factors. The crop with highest damage was cocoa (63.9% plants affected by black pod disease) and we also recorded a widespread incidence of the vascular streak dieback (19.4% plants affected). Fusarium stem rot was the second highest damage in crops, with 30.0% of vanilla plants infected. Fusarium wilt affected 10.2% of banana plants and the coffee berry borer affected 9.7% of coffee plants. There were no differences in pest and pathogen incidence between community forest and polyculture, thus insecticides and fungicides had little to no impact on crop infestations. An increased crop richness favoured a reduction in some insect pests, suggesting a potential increase in natural pest control. Since insecticides and fungicides do not seem to have a significant impact in reducing pest and pathogen infestation (probably due to genetic variability and resistance of pests and pathogens), the most effective Indigenous practice is to diversify profits from different crops, thus buffering the negative effects of market fluctuations and yield loss.

## Graphical abstract

The crop with the highest damages was cocoa (63.9% plants affected by black pod disease), followed by Fusarium stem rot was the second highest damage in crops (30.0% of vanilla plants infected). Insecticides and fungicides do not have a significant impact in reducing pest and pathogen infestation. The most effective indigenous practice is to diversify profits from different crops, thus buffering negative effects of market fluctuations and yield loss.

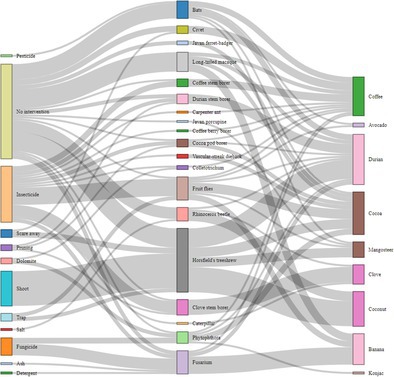

## 1 Introduction

### 1.1 Indigenous Communities and Agroforestry Systems

Agriculture is the largest driver of global land use, especially in the tropics, contributing significantly to deforestation, biodiversity loss, and climate change (Pendrill et al. 2022). Conventional farming systems typically support low faunal diversity due to their use of agrochemicals, simplified ecosystems, and low floral diversity (Kremen and Miles 2012). Indigenous farming practices (apart from destructive ones such as slash‐and‐burn; Altieri 2004) are known for their lower impact on biodiversity and for having a relatively high level of agrodiversity (Brookfield and Padoch 1994; Hamadani et al. 2021). They are considered pivotal for maintaining biodiversity in agricultural lands (Sharma et al. 2020). It is important to link traditional practices with more recent innovations to promote long‐term sustainability of lands (Altieri 2004). Agroforestry (i.e., integrating trees and diverse crops within agricultural landscapes) is an area where Indigenous Knowledge and innovation can be integrated in view of food security and habitat sustainability (Gonçalves et al. 2021; Budiadi et al. 2025). By establishing partnerships between farmer communities and innovators and promoting traditional agricultural knowledge, agroforestry not only can restore biodiversity but also empowers farmers to create more sustainable, climate‐resilient systems (Nair 2014). Collaborations between researchers, governments, and farmer communities allow for the co‐creation of agricultural systems that balance ecological health and economic productivity, providing a realistic solution to biodiversity loss and environmental degradation (Tan et al. 2022).

Higher biodiversity within agroforests can enhance ecosystem services and reduce the reliance on agrochemical use (Kumar and Nair 2020). For example, diverse plant communities improve soil fertility, nutrient cycling, and water retention, potentially leading to increased productivity (Maestre et al. 2012). The relationship between crop yields and plant diversity, however, is not always clear‐cut and depends on the types of plants and crops involved (Verdu et al. 2010). Additionally, biodiversity enhances pest regulation and pollination services due to a greater abundance of natural predators and diverse flora, which can ultimately increase crop yields (Cardinale et al. 2012; Wan et al. 2018). Similarly, ecosystems with greater species diversity usually exhibit higher resilience to environmental changes, helping maintain critical functions such as carbon storage and habitat provision (Isbell et al. 2017). Furthermore, biodiverse ecosystems experience smaller temperature fluctuations, making them potentially more resilient to climate change and better suited to supporting species amid rising temperatures (Tilman et al. 2014). This resilience is crucial for the future of agriculture, as commercial farms may struggle to adapt to changing environmental conditions.

### 1.2 Context

In this study, we focused on pests and pathogens affecting four major cash crops in Indonesia: banana, cocoa, coffee, and vanilla. Indonesia is the third‐largest producer (in terms of production value) of cocoa, which is cultivated across around 1.4 million hectares, but its production has declined by 50% in the last decade due to factors such as pest infestations, disease outbreaks, and soil fertility loss (Statista 2025; Fahmid et al. 2018). Indonesia ranks as the second‐largest coffee producer in terms of land area, with approximately 1.3 million hectares cultivated, and an increase in coffee production of about 25% in the last 10 years (Statista 2025). That said, coffee production has been predicted to decline in the future due to climate change and increased pest incidence (Jawo et al. 2023). Notably, most (> 95%) of the production of coffee and cocoa in Indonesia is done at a smallholder scale (Statista 2025). Indonesia is also the world's second‐largest vanilla producer, reaching its peak production in 2011. However, production steadily declined until 2018, likely due to export challenges and environmental changes (Nurjati 2019). Indonesia is the third‐largest banana producer, and its production increased in the last decade by ~40% (Statista 2025). Climate change has been indicated to potentially increase pathogen incidence, thus posing a potential threat to banana production in the future (Alkhalifah et al. 2023). Addressing the actual or potential decline in productivity due to an increase in pest and pathogen incidence is essential.

### 1.3 Pests and Pathogens in Banana, Cocoa, Coffee, and Vanilla

The cocoa pod borer (*Conopomorpha cramerella*) is a moth species and a major pest causing severe damage to cacao crops. The larvae burrow into cacao pods, consuming the fruit and causing discolouration, shrivelling, and rotting. Infested pods develop large exit holes, leading to total crop loss. Infestations can result in yield losses ranging from 20% to 100% and can also facilitate the spread of fungal pathogens, such as the pathogen causing black pod disease (*Phytophthora* spp.) (Ploetz 2000). Cocoa pod borer abundance is shown to be more prevalent in highly shaded farms compared to less shaded ones (Azhar and Long 2013), and this prompted a shift toward cultivating cacao in lower‐shade environments (Yamada and Nishida 2009). The black pod disease, however, does not seem to be influenced by the shade tree cover (Gidoin et al. 2014). The vascular streak dieback caused by the fungal pathogen *Ceratobasidium theobromae* has also been shown to affect cocoa plants and is considered to be an emerging disease (Ramírez‐Camejo et al. 2025). Small mammals such as squirrels and treeshrews are frequently attracted to cacao farms, particularly those with dense shade cover and complex vegetation, which provide ideal shelter and nesting sites and they can cause substantial damage to cacao pods (Molina and Mazón 2022; Vansynghel et al. 2022).

The coffee berry borer (CBB; *Hypothenemus hampei*) is a beetle species that preys on coffee beans. Female CBB create holes at the base of coffee berries, burrowing inside and hollowing them out (Vega et al. 2015). They also disperse pathogens (*Colletotrichum* spp.) that cause the coffee berry disease and affect nearby berries (Serrato‐Diaz et al. 2020; Manson et al. 2022). CBB cause a global economic loss of half a billion US‐dollar per year (Escobar‐Ramírez et al. 2019). CBB populations are usually lower in heterogeneous habitats (e.g., shaded coffee gardens) compared to less complex habitats (e.g., unshaded plantations) (Teodoro et al. 2009; Manson et al. 2022; Vilchez‐Mendoza et al. 2022). This decrease is likely due to the ability of shade agroecosystems to support beneficial arthropods, which act as natural predators, thereby enhancing biological control (Johnson et al. 2020; Manson et al. 2024). This reduction is also attributed to the cooler temperatures in shaded environments, which suppress pest activity. Furthermore, shaded coffee systems in some contexts demonstrated higher yields, potentially due to improved soil conditions and water cycles (e.g., Jaramillo et al. 2009), despite other situations where a neutral or negative effect of shade on yields has been shown (Piato et al. 2020; Lalani et al. 2024).

Fusarium wilt, caused by *Fusarium oxysporum f*. sp. *cubense* (Foc) threatens banana plantations with devastating vascular damage, while Fusarium stem rot, induced by *Fusarium oxysporum* f. sp. *vanillae* (Fov), imperils vanilla yields through stem dieback and plant mortality (Pegg et al. 2019). In bananas, Fusarium wilt manifests as yellowing leaves, pseudo stem splitting, and eventual plant collapse, often leading to complete crop failure (Ghag et al. 2015). In vanilla, Fusarium stem rot is apparent as wilting, discolouration, and dieback, reducing pod production and threatening plant viability (Koyyappurath et al. 2016). Their success as phytopathogens derives from their biological adaptability, including the formation of chlamydospores‐dormant, resistant structures that enable long‐term survival in soil, and their ability to colonise plant vascular systems, disrupting water and nutrient transport (Orr and Nelson 2018). Dense canopies elevate humidity and soil moisture while reducing temperature fluctuations, conditions that favour fungal growth and spore dispersal (Yang et al. 2022). Conversely, sparse canopies may expose crops to greater sunlight and drier conditions, potentially suppressing pathogen activity (Zakaria 2023). Polyculture systems, with their diverse crop assemblages, may dilute pathogen pressure by reducing host density or fostering antagonistic microbial communities in the soil (Chavez et al. 2024). For instance, intercropping bananas with species like pineapple or legumes could disrupt Fusarium transmission cycles (Were et al. 2023). Vanilla is also susceptible to multiple insect pests, including the vanilla weevil and vanilla thrips. The vanilla weevil primarily targets the stems and vines, boring into them and feeding on the plant's structural tissue (Vanitha et al. 2011). This damage leads to wilting and browning of the vine and leaves, with visible entry holes where the pest has burrowed. Additionally, the weevil lays its eggs inside the vanilla crop, further compromising plant health (Varadarasan et al. 2002). In contrast, vanilla thrips primarily affect the leaves and flowers, feeding on plant juices and causing leaf deformation and, in some cases, flower abortion. Infestations leave distinct silver streaks on the leaves, creating a different pattern of damage compared to the weevil (Devasahayam et al. 2022).

### 1.4 Study Aims

This study aims to understand which factors affect pest and pathogen incidence in agroforestry systems in Bali, Indonesia, and how Indigenous communities manage to limit their incidence. Balinese culture embraces mixed farming systems that integrate traditional knowledge and agricultural innovation (Wibowo et al. 2024). Agroforestry systems in Bali also have an important cultural component since products are used for religious offerings, festivals, and culinary traditions (Adiputra 2018). Thus, understanding how to limit pests and pathogen incidence in line with local practices is not only important to guarantee the sustainability of land use but also the sustainability of the Indigenous culture.

We expect that croplands containing a high shade cover will provide a more complex habitat for species, so a lower proportion of crop pests, specifically invertebrates such as coffee borers and insect pests of vanilla, as there are more natural predators. However, it might provide more resources for smaller mammal pests and fungal infestations (which increase with increased moisture levels). At the same time, a diverse crop assemblage can reduce host density or foster antagonistic microbial communities in the soil, limiting pathogen transmission. We also expect that the agroforestry systems where agrochemicals are used with no limitations (i.e., polycultures) have fewer pests and pathogens than the community forest (where the use of agrochemicals is precluded by social rules).

## 2 Methods

### 2.1 Study Site and Environmental Context

**FIGURE 1:**
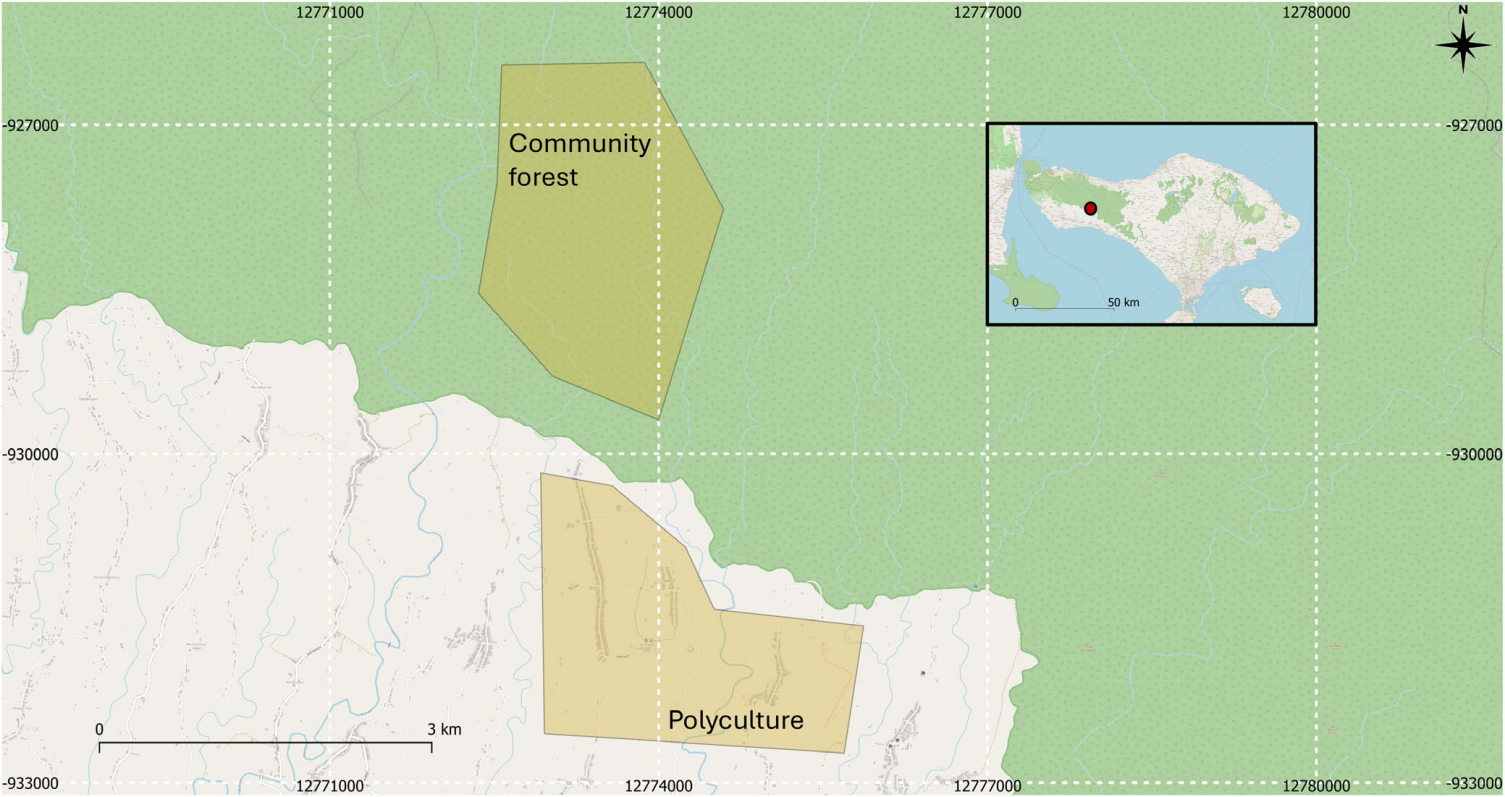
Map of the study area in Bali, Indonesia. The polyculture system included 51 plots from two villages (Yeh Buah and Kedisan, included in the polygon); the community forest included 49 plots. Polygons indicate the extension of land that includes the plots in the two systems. The green shading indicates mature forests, which include community forests.

Our study was conducted in August 2024 and May–June 2025 in the villages of Yeh Buah and Kedisan, Jembrana Regency, Bali, Indonesia. The study site consists of two distinct agroforestry systems: polyculture agroforests and community‐based forests (Figure 1). In polyculture agroforests, farmers cultivate multiple crops including clove ( *Syzygium aromaticum* ) and coconut ( *Cocos nucifera* ) (Campera et al. 2024). They are located closer to the village, making them more accessible and subject to increased human activity and potential environmental disturbances. In contrast, community‐based forest plots are cultivated within forest land managed by the provincial forestry authority, Kesatuan Pengelolaan Hutan Bali Barat (KPH). The Indigenous community is allowed to grow crops under the native forest, but they are regulated by several social conventions that prohibit the cultivation of crops that are considered not suitable to grow under the forest and potentially invasive (e.g., clove and coconut) (Campera et al. 2024). Farmers hold lands both in the agroforestry and community forest (concessed by KPH). Furthermore, in community forests, farmers are not allowed to use agrochemicals, thus do not have chemical measures to contain pests and pathogens. The terrain is steeper and more remote compared to polyculture agroforests, making accessibility more challenging.

To investigate how habitat variables influence pest incidence, we established 100 study plots (51 in polyculture systems and 49 in the community‐based forest), each measuring 25 × 25 m^2^. Plots were spaced approximately 50 m apart to account for ecosystem and community differences. We followed recommendations and approval from local leaders to decide where to place plots. The study area's elevation ranges from 220 m to 480 m above sea level, with an annual precipitation of approximately 1000 mm, with a wetter season (October–April) and a drier season (May–September).

### 2.2 Data Collection Methods

Within each plot, we focused on four primary crops: banana (*Musa* spp.), coffee ( *Coffea canephora* ), cacao ( *Theobroma cacao* ), and vanilla ( *Vanilla planifolia* ), due to initial discussions with local farmers regarding significant pest and pathogen issues and their important role in the local economies. A maximum of five plants per species were sampled in each plot. A random walk‐on‐clock hands method was employed to minimise sampling bias and ensure representative coverage. A member of the team stood at the plot's centre, identified using a georeferenced centre point, and used a wristwatch with an analogue face to determine direction. The ‘seconds’ hand position (e.g., pointing to 15 s = 90° east) influenced movement towards the nearest plant within a 5 m radius, sampling a maximum of plants per crop. This ensured a representative sample size for each plot, reducing the sampling effort. This is to allow for sampling multiple crops and species during their fruiting period and before harvest time. In plots with fewer than five specimens present to sample (a minimum of one), the maximum number present was recorded.

**FIGURE 2:**
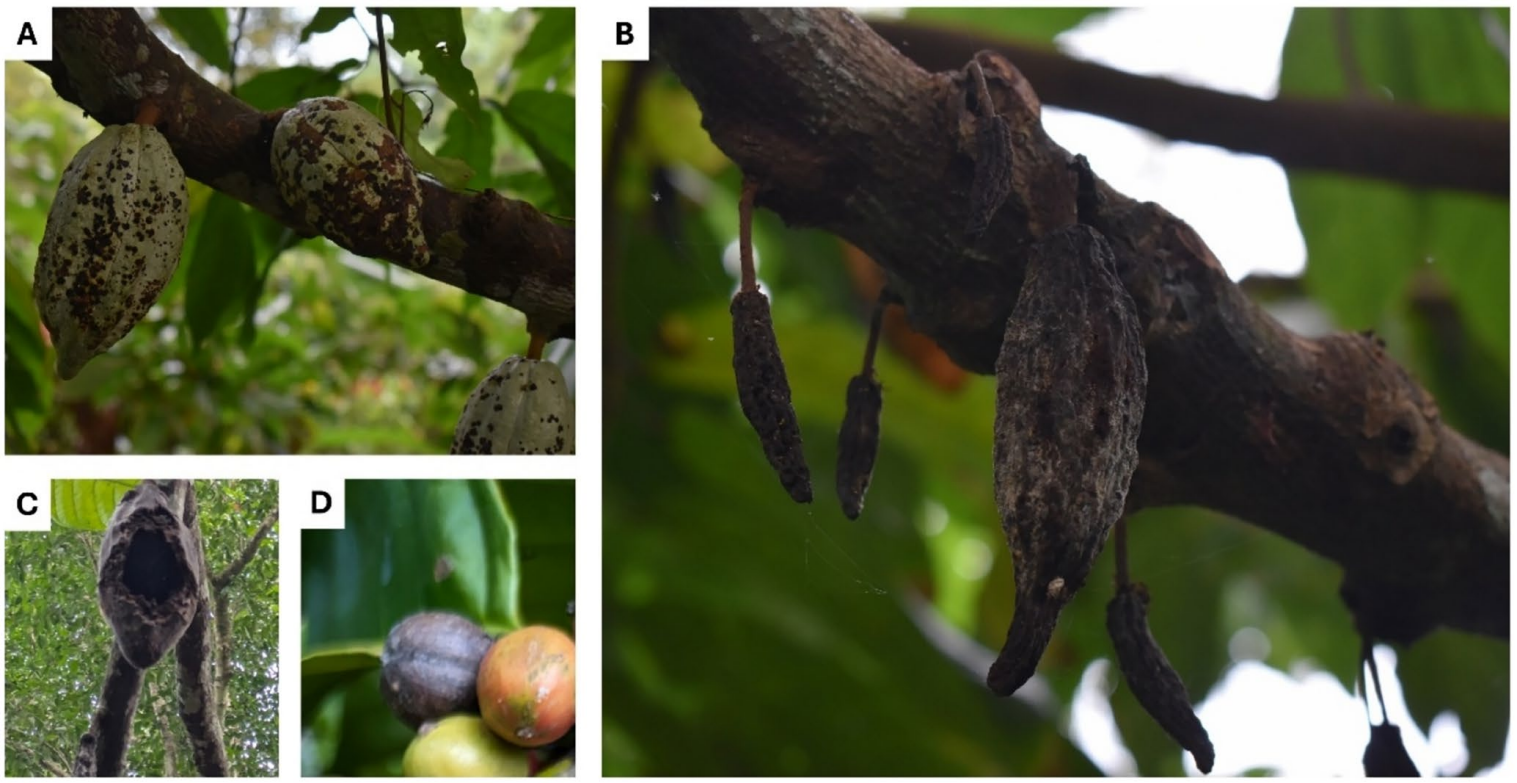
Signs of pests and pathogens in cocoa and coffee. The black pod disease can be spotted at early stages (A) as well as at an advanced stage (B). Cocoa berries can also have a fungal infestation after being eaten by Horsfield's treeshrew *Tupaia javanica* (C). Coffee berries can show signs of coffee berry borer damage associated with secondary fungal infections (D).

Cacao pest infestations were assessed based on visible signs of fruit stem damage. A fruit was considered infested by herbivore pests if it displayed clear entry holes or signs of consumption. Potential cacao pests included both insects (cocoa pod borer) and larger vertebrates (Horsfield's treeshrew *Tupaia javanica* ) (Mohan et al. 2022). Infestation severity was recorded using a scale of 1–10, based on the proportion of branches bearing damaged cacao pods. Severely infested fruits were typically hollowed out, leading to rot and secondary fungal infections. Fungal infestations were assessed in a similar way, but in addition to fruit damages (black pod disease), stem damages (vascular streak dieback) were assessed (Figure 2).

Coffee pest assessments focused exclusively on coffee berries, as these were the primary targets of infestation. The main pest of interest was the coffee berry borer (*Hypothenemus hampei*), which bores into the bottom of the coffee berry, creating entry holes and hollowing out the inside. This damage often leads to secondary fungal infections (*Colletotrichum* spp.), causing additional crop loss (Waller et al. 2007). The presence of affected berries was visually assessed, and each plant was assigned an infestation score from 0 to 10, based on the proportion of branches bearing coffee berry borer‐infested berries (0 = all branches unaffected; 10 = all branches affected) following the method outlined by Manson et al. (2022). Other coffee pests, such as common palm civets ( *Paradoxurus hermaphroditus* ), were not recorded due to their nocturnal behavior and lack of immediate visible signs of damage. The coffee leaf rust is also present in the area but not assessed here as it was not considered a major pathogen by the Indigenous community.

**FIGURE 3:**
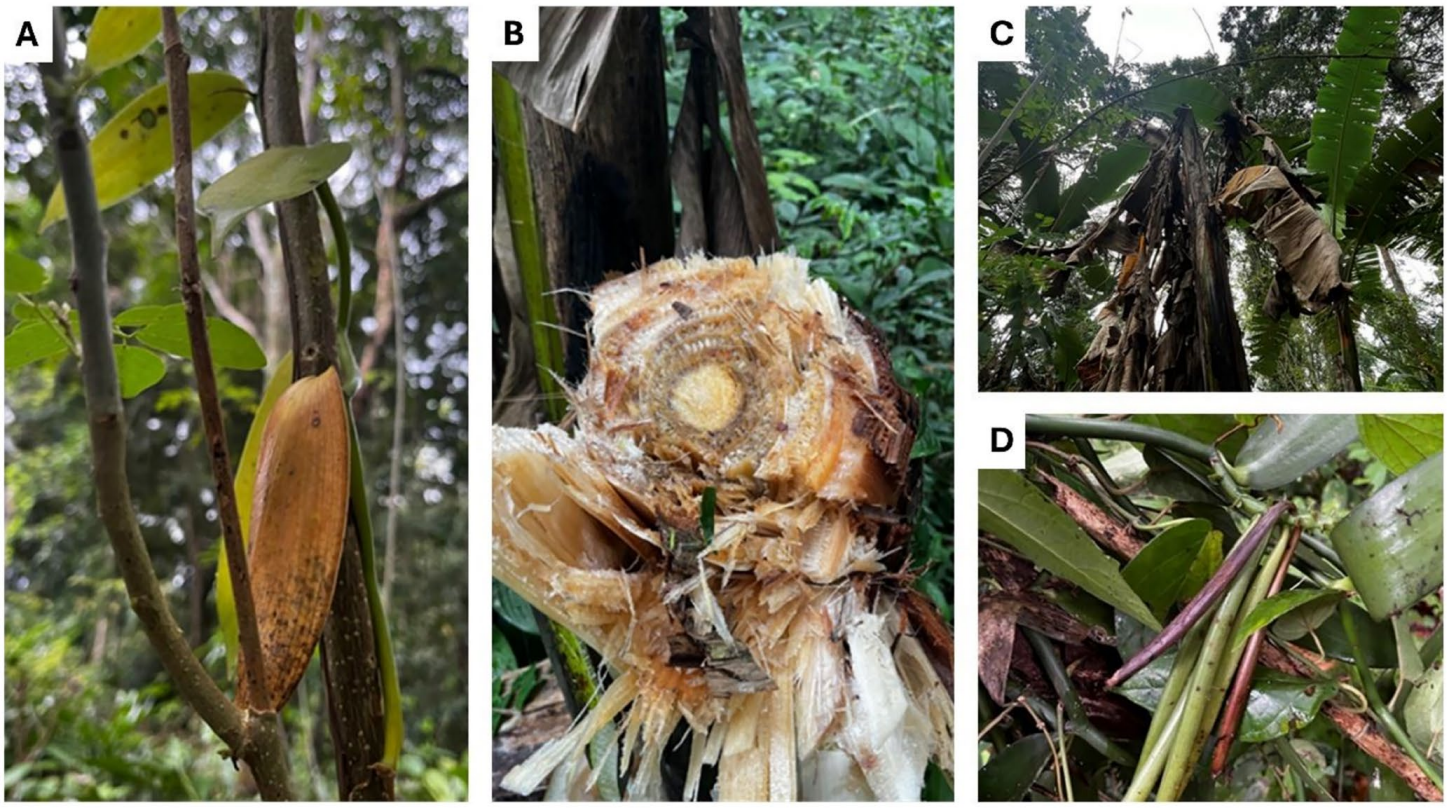
Signs of infestation of *Fusarium oxysporum* in vanilla (Fov) and banana (Foc). Fov is evident from fungal growth and yellow discoloration of leaves (A) and pods (D). Foc is leaving brown discoloration and signs of leaf wilting (B, C). Pictures are taken by the authors in the study area.

Vanilla infestations were assessed at the fruit and/or stem level. Pest damage was recorded based on direct observations of herbivory, including the presence of chewing damage, boreholes, and pest species such as caterpillars, beetles, and ants. Fusarium presence was assessed visually, focusing on symptoms. Samples were classified as infected if they displayed obvious symptoms such as the following: for bananas, Fusarium wilt symptoms included leaf yellowing, wilting, and pseudostem splitting, while vanilla orchids were inspected for stem rot, discoloration, and fungal growth on stems or roots (Figure 3). Since the infestations for vanilla and banana affect the majority of the plant, a binary presence/absence system was used instead of a proportional infestation score.

In addition to crop pest data, we collected ecological data to assess ecosystem complexity, including measurements of shade cover and crop species richness (i.e., number of crop species). To determine canopy cover, we used the Canopeo application, which calculates the proportion of shaded area from photographs (Patrignani and Ochsner 2015). Within each plot, we took four random and independent canopy photographs, ensuring a minimum distance of 5 m between photo capture points and a 5‐m minimum distance from the edge of the field to avoid edge effects Photographs were taken at approximately 5 m intervals, and care was taken to ensure that crop leaves, such as banana leaves, did not obstruct the view or bias the calculation of tree shade cover. The mean shade cover value for each plot was calculated from these photographs (Campera et al. 2024).

We also collected data on the measures taken to mitigate pests and pathogens by interviewing 35 farmers from the community in November 2024. The interview included other sections and lasted around 15–20 min, but for this paper we only present the relevant findings that are part of the following questions: (1) How many different types of crops do you grow? Which ones? (2) What are the major pests and pathogens that affect your crops? What type? (3) What are the pest and pathogen control methods you use?

### 2.3 Ethics and Permission

Our research in Indonesia is underpinned by a Memorandum of Understanding between Oxford Brookes University and Universitas Warmadewa (Denpasar). Indonesia's National Research and Innovation Agency granted permission to conduct the research (161/SIP/IV/FR/4/2024; 168/SIP/IV/FR/4/2024; 171/SIP/IV/FR/4/2024). Data collection on human participants was approved by the Oxford Brookes University Ethics Committee (UREC Registration No: L24357).

### 2.4 Statistical Analysis Methods

We report the percentages of plants infected by pests and pathogens for each crop, as well as the average score of infection for the crops (i.e., coffee and cocoa) for which we gave a 0–10 score. From the scores we then calculated 0–1 scores (indicating the average percentage of plant infected) for each type of pest of pathogen and used that for further analyses. We used Generalised Additive Models via R version 4.3.1 to test whether crop richness (a measure of the complexity of the cropland), canopy cover (a measure of the light received by the crops) and agroforestry type (i.e., whether the plot was located in the community‐based forest or in the polyculture system) were influencing the incidence of pests and pathogens. We added a smoothing term for canopy cover since we expected a non‐linear relationship. We ran the models via the function *gam* and checked the model fit via the function *appraise* in the package *mgcv* (Wood and Wood 2015). We tested the relevant fit families, i.e., Gaussian, Tweedie (tw), quasipoisson, quasibinomial, and link functions and selected the model with the best fit based on the *appraise* output. We selected the following families: tw for CBB infestation; gaussian (log link) for Fov infestation; quasipoisson for cocoa herbivore, vanilla herbivores and Foc infestation; gaussian for cocoa fungal infestation. We added the number of plants censused in the plots as weight in the models to account for the uneven sampling effort by plot. We plotted the significant relationship and trends for the relationship with canopy cover and infestation via the *draw* function in *gratia* package (Simpson 2024). We used the function *sankeyNetwork* in the package *networkD3* to create the Sankey diagram to represent the responses of farmers to the questions in the interview (Lim et al. 2025). Interviews were first coded into pests and pathogens affecting crops, as well as the control methods used. Farmers provided up to five responses. We then created two columns with sources (pests/pathogens or control methods) and targets (crops or pests/pathogens), with a value corresponding to the number of farmers who mentioned the link. This information was used to create the Sankey diagram.

## 3 Results

### 3.1 Main Pests and Pathogens

In total, we surveyed 412 banana plants, 318 coffee plants, 263 cocoa plants, and 180 vanilla plants. Cacao was present in 65 plots (36 community‐based forest plots and 29 polyculture plots), coffee was present in 74 plots (40 community‐based forest plots and 34 polyculture plots), vanilla in 41 plots (32 community‐based plots and 9 polyculture plots), and banana in 87 plots (41 community‐based plots and 46 polyculture plots). Cocoa plants showed an average score of fungal infestation of 0.41 (i.e., on average, 41% of the plant is affected), with 63.9% plants affected by black pod disease and 19.4% affected by vascular streak dieback. Cocoa plants showed an average score of herbivore pests of 0.30 (i.e., on average, 30% of the plant is affected), with most of the damage being done by Horsfield's treeshrew, affecting 49.0% of cocoa plants. The cocoa pod borer was not a major pest in the area, only affecting 4.6% of cocoa plants. Signs of *Helopeltis* spp. infestation have been recorded on three cocoa plants from three different plots. 30.0% of vanilla plants were infected by Fov, and 27.0% showed signs of herbivory. 10.2% of banana plants were infected by Foc. Coffee plants had the lowest infection score (mean: 0.04; i.e., on average, 4% of the plant is affected), with 9.7% of plants affected by CBB.

### 3.2 Factors Affecting Pests and Pathogens

**FIGURE 4:**
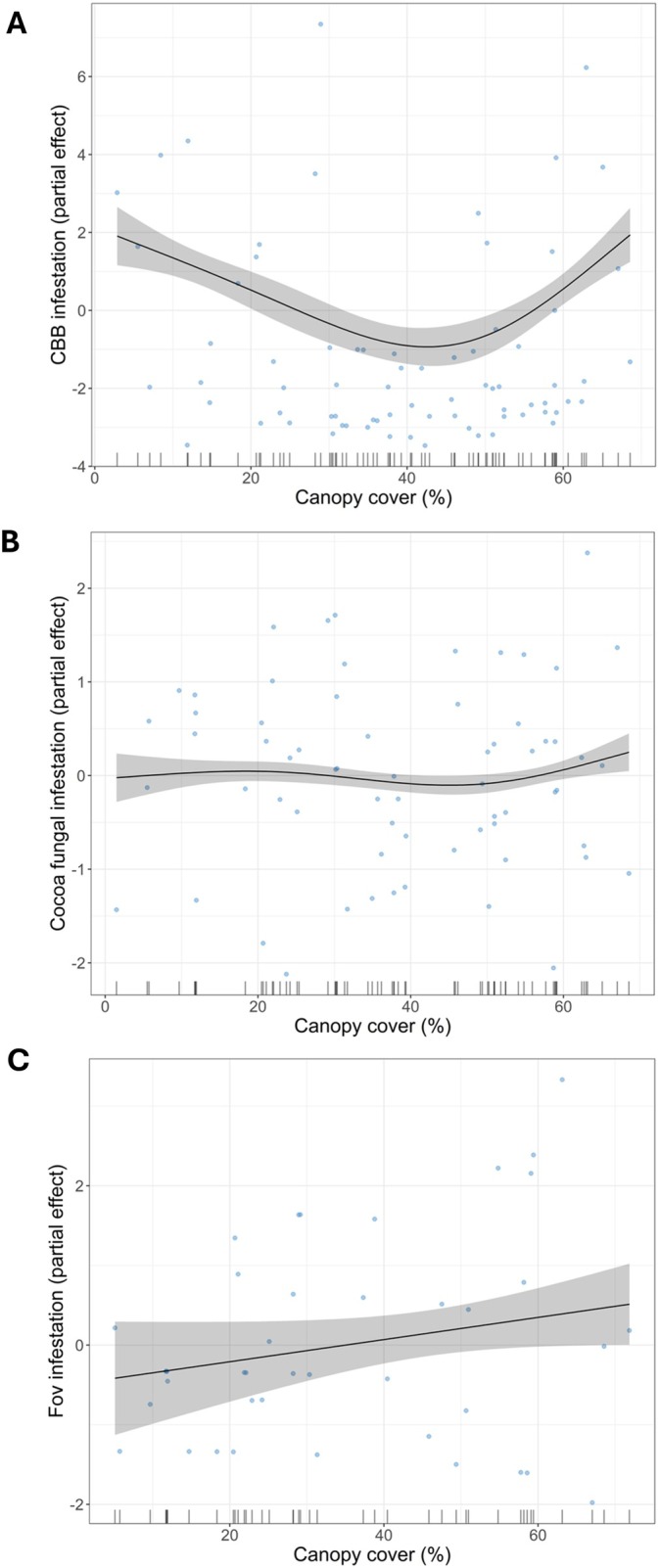
Relationship between canopy cover and pest/pathogen infestation. (A) Significant (*P* < 0.001) effect of canopy cover on CBB infestation, which decreases at intermediate canopy cover. Reference value indicates 4.12% of infestation; (B) trend (*P* = 0.082) towards a significant effect of canopy cover on cocoa fungal infestation, with values of canopy cover > 60% being above the reference value. Reference value indicates 40.69% of infestation; (C) Trend (*P* = 0.098) towards a significant positive effect of canopy cover on Fov infestation. Reference value indicates 29.74% of infestation. Full model outputs are in Table 1.

CBB infestation on coffee berries was reduced by an increase in richness of crops (*P* = 0.007) and intermediate levels (30%–50%) of shade cover (Table 1, Figure 4). An increase in richness of crops also determined a reduction in signs of herbivory on vanilla plants (*P* = 0.027). High canopy cover (above 60%) tended to induce an increase in fungal infestation in cocoa (*P* = 0.082) and vanilla (*P* = 0.098) (Figure 4), but with only a trend towards significance. There was no effect of the agroforestry type (i.e., community‐based forest vs. polyculture) on the infestation of pests and pathogens (Table 1).

**TABLE 1:** Results of Generalised Additive Models to explain determinants of pest and pathogen incidence. Edf: Effective degrees of freedom.

Response	Predictor	Estimate	Std Error	*t*‐value	Smooth term	
					edf	*F*‐value
CBB infestation	Intercept	−2.637	0.590	−4.473***		
	Richness of crops	−0.265	0.095	−2.784**		
	Canopy cover (%)				2.81	17.47***
	Agroforestry type	0.286	0.318	0.897		
Cocoa fungal infestation	Intercept	0.340	0.136	2.501*		
	Richness of crops	0.014	0.018	0.461		
	Canopy cover (%)				2.75	2.12
	Agroforestry type	−0.013	0.077	−0.164		
Cocoa herbivores	Intercept	−1.174	0.418	−2.809**		
	Richness of crops	−0.004	0.057	−0.066		
	Canopy cover (%)				1.00	0.01
	Agroforestry type	0.001	0.240	0.002		
Foc infestation	Intercept	−2.302	0.816	−2.821**		
	Richness of crops	−0.057	0.122	−0.467		
	Canopy cover (%)				1.00	2.29
	Agroforestry type	0.526	0.445	1.182		
Fov infestation	Intercept	−1.173	0.644	−1.822		
	Richness of crops	0.002	0.127	0.013		
	Canopy cover (%)				1.00	2.91
	Agroforestry type	−0.111	0.331	0.739		
Vanilla herbivores	Intercept	−0.342	0.864	−0.396		
	Richness of crops	−0.369	0.159	−2.324*		
	Canopy cover (%)				1.00	0.15
	Agroforestry type	0.577	0.662	0.872		

* *P* < 0.05.

** *P* < 0.01.

*** *P* < 0.001.

### 3.3 Local Measures to Control Pests and Pathogens

**FIGURE 5:**
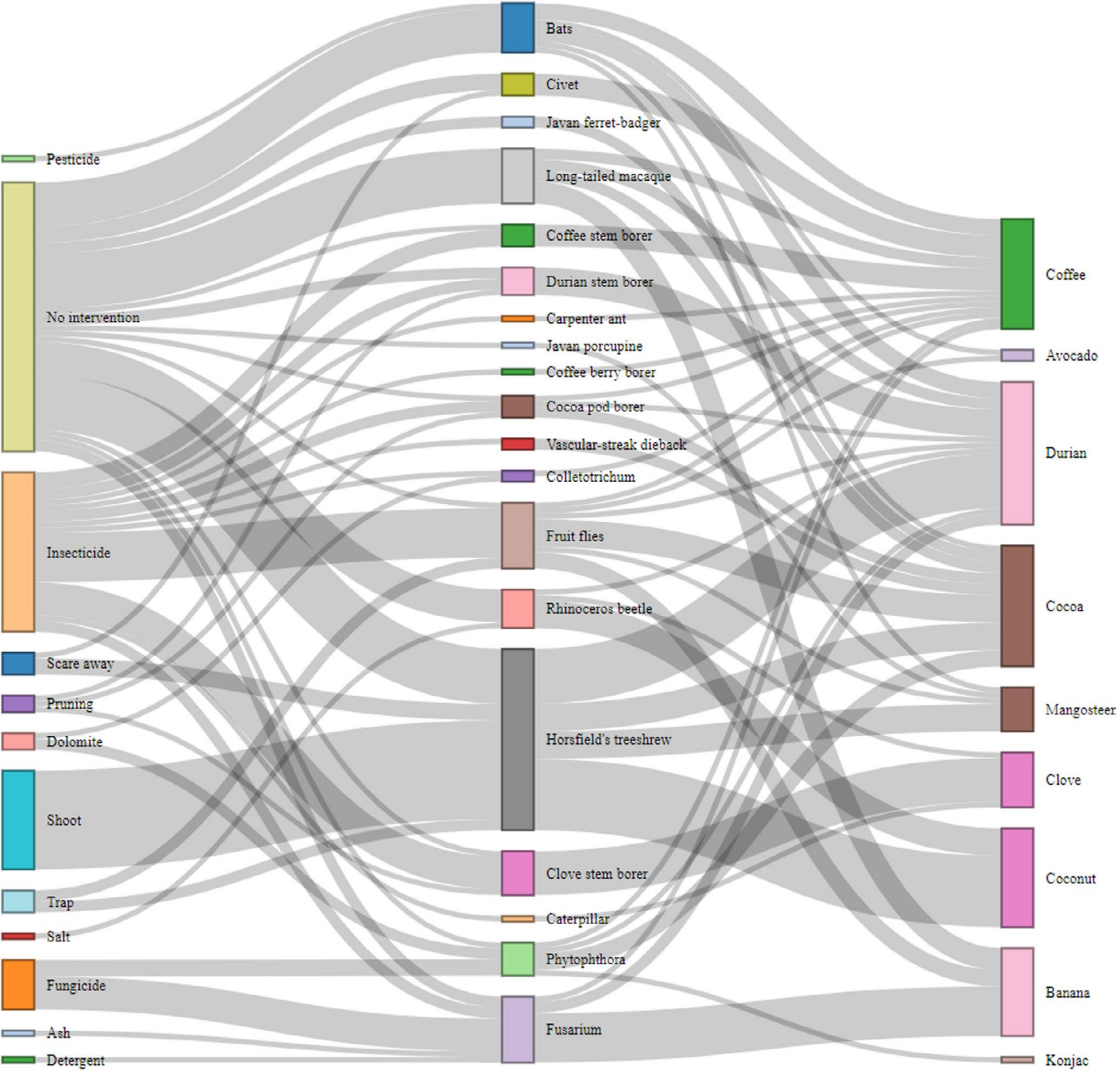
Sankey diagram showing the methods used to mitigate pests and pathogens in crops by farmers in Yeh Buah community, Bali, Indonesia. The weight of the links and items corresponds to the number of farmers who reported that specific link. The specific number of farmers who reported each link can be found in the raw data associated with the paper.

Coffee, cocoa, and banana were reported as important crops affected by pests and pathogens in the area, together with durian and coconut. Vanilla was not mentioned during the interviews. Fusarium was not only reported as a threat to banana, but also to cocoa and coffee. Vertebrates are considered a major pest in the area (82.8% of farmers). Horsfield's treeshrew was the most reported pest (57.1%), and it is the only vertebrate for which invasive measures (i.e., shooting) were reported (17.1%). It was suggested to create issues not only for cocoa but also for coconut, durian and mangosteen production. Other vertebrates were less reported: bats and macaques (17.1%), civets (11.4%), Javan ferret‐badger (5.7%), and Javan porcupine (2.9%). No intervention was the most common action (39.8% of links), while insecticides or fungicides were used in 29.3% of the cases. Non‐chemical alternatives were used, although rarely: dolomite is used by two farmers to control coffee berry disease and black pod disease; ash is used by one farmer to control Fusarium; salt is used by one farmer to control rhinoceros beetles that are major pests for coconut. While one respondent suggested that carpenter ants (*Camponotus* spp.) are pests and indicated the use of insecticides to control them, other respondents suggested that there is potentially a beneficial role of carpenter ants in reducing CBB infestation (Figure 5).

## 4 Discussion

We found no difference between community‐based forests and polyculture agroforests in terms of incidence of pests and pathogens, suggesting that the use of agrochemicals might not bring a significant reduction in pests and pathogens (considering that in community forests insecticides and fungicides were not allowed). Conversely, canopy cover and richness of crops have a significant effect on some pests and pathogens, suggesting that cropland composition and structure are more important to limit infestation (Sollai and Solari 2022). This is likely to be due to an increase in ecosystem services (including natural pest control) and competition. Having crops under a native forest canopy can also favour carbon sequestration, and having large native trees does not reduce crop productivity (Alexander et al. 2025). Tree and crop diversity can also enhance overall crop production by improving soil health and fostering a more environmentally resilient farming system (Gurr et al. 2003; Chavez et al. 2024). This is an incentive for farmers to increase crop and native tree diversification on their farms, rather than viewing it solely as a conservation benefit. In addition, cultural practices can be favoured since sacred patches of forests are preserved, and this can bring long‐term benefits such as climate change mitigation (Maru et al. 2023). That said, having too dense canopies (above 60%) seems to favour excessive fungal growth, as also shown elsewhere (e.g., Molina‐Monteleón et al. 2024).

Fungal infestations on cocoa plants were the major issue in the area, with the main threat represented by the black pod disease. The vascular streak dieback seems to be an emerging disease in the region considering that this is the first published evidence of the disease in Bali and that new symptoms have been found in Indonesia, suggesting genetic variation of the pathogen (Bryceson et al. 2023). Shade cover had a weak effect on pathogen infestation, although there was a tendency for plots with very dense canopies (> 60%) to have higher infestation. But we found no evidence that sun‐exposed plants are less likely to be infected, contrary to previous research (Azhar and Long 2013). The characteristics of the vegetation structure and vegetation composition might have a more important effect than shading (Gidoin et al. 2014; Manson et al. 2024). Based on the lack of significant differences between the two agroforestry systems, the use of fungicide does not seem to help reduce the pathogen infestation possibly due to the genetic variation and resistance of the pathogens.

Crop richness did not have a significant effect on Fusarium infestation. This might be due to a combination of antagonistic competition with soil microbes (e.g., *Trichoderma*; Xiong et al. 2016) and the dilution effect hypothesis (Trenbath 1993), which suggests that diverse plant assemblages reduce pathogen pressure by lowering host density. Non‐host species like clove and teak in polyculture plots could disrupt Fov and Foc cycles, while rooting exudates from coffee or bamboo trees might foster antagonistic soil microbes, such as *Trichoderma*, that suppress Fusarium (Todorović et al. 2023). Other factors such as the proportion of herbaceous crops could have also affected the presence of Fusarium by elevating humidity or organic matter inputs. Some herbaceous crops can also release antifungal isothiocyanates, which can be useful to naturally decrease Fusarium infestation (Muturi et al. 2024). There is evidence that sequential diversity rather than intercropping can disrupt Fusarium incidence (Fan et al. 2022). Fusarium has also been shown genetic plasticity and resistance to conventional methods (Zhou et al. 2023). There is thus the need to do more testing on the factors affecting Foc and Fov infestation, seeking optimal management techniques for their natural reduction.

Vanilla is the crop with the highest potential as a cash crop, with prices up to US$600/kg (Munshi 2020), and it was a major income for farmers in Yeh Buah in 2023 (Campera et al. 2024). Vanilla is also prone to different pests and pathogens, although relatively resistant compared to other crops (Wahyudi et al. 2023). It is thus interesting that farmers did not mention vanilla in their interviews. This can be explained by the fact that vanilla prices are fluctuating (following the global market) since there is no stocking facility for vanilla in the region. Selling prices for vanilla in 2024 were relatively low, thus many farmers decided not to invest in vanilla (given that it is the crop that requires the highest input of time and labour due to the need to hand pollinate flowers; Munarso et al. 2024). As a result, the production of vanilla in the two villages was very low in 2025, and farmers did not invest in vanilla cultivation at the time of the interviews.

Insect pests of coffee and vanilla were reduced by having a diverse cropland, in line with other findings in Indonesia and other areas (Munir and Bashir 2019; Campera et al. 2024). In addition, we found a limited incidence of the two main insect pests of cocoa: cocoa pod borer and *Helopeltis* spp. (Azizah et al. 2025). The cocoa pod borer was also not a major pest in the area, compared to other areas where cocoa is cultivated in less complex habitats (Akesse‐Ransford et al. 2021). The impact of these two pests, however, might have been underestimated since it is often during harvesting, when the fruit is shelled, that the damage becomes visible (Valenzuela et al. 2014). It is also important to consider the composition of trees, as that can have an impact on natural pest control and insect diversity (Somarriba et al. 2024). Lower shade croplands may also have a higher insect pest incidence since insectivore birds are sensitive to habitat disturbances (Imron et al. 2022). Shade can also regulate pest populations by influencing microclimate conditions such as temperature and humidity (Liebig et al. 2018). The results of our study support the literature that coffee grown in biodiverse systems will experience less pest incidence and therefore increased yields (Cardinale et al. 2012; Manson et al. 2022). Although intermediate shade (30%–50%) seems to favour the reduction in CBB infestation, habitats with very dense canopies might not be efficient enough to limit CBB infestation (Oliva et al. 2023).

From the results, it is evident that vertebrate pests are a major issue in the area. Vertebrate pests of crops (e.g., Horsfield's treeshrew but also common palm civets and long‐tailed macaques *Macaca fascicularis* ) prefer diverse habitats and dense canopies that offer food options and ease of movement (Campera et al. 2021; Manson et al. 2024), bringing potential issues and conflicts for the Indigenous community. The presence of diverse crops, particularly fruit trees, can also attract rodents (Caudill et al. 2014; Campera et al. 2021). While for Horsfield's treeshrew farmers clearly indicated invasive methods (i.e., shooting) being used, for other species (especially macaques) the consensus by Indigenous communities is to not intervene. Macaques have a spiritual connotation in Bali since they are associated with the monkey god “Hanuman”, which is revered in Hinduism (Fuentes 2010; Olthoff et al. 2026). Macaques are not necessarily sacred to all Balinese, as in some villages there are more conflicts emerging if the macaques are particularly destructive (Fuentes 2010) and in some of the more touristic cities their skulls are openly offered for sale (Chavez and Nijman 2024; Chavez et al. 2025). Civets are also known to be traded in Bali, mostly for civet coffee farming (Nijman et al. 2024). Diverse farms might provide alternative food sources for vertebrate pests, thereby reducing their impact on cash crops.

From the results, it is evident that other factors might explain the incidence of pests and pathogens in the area. A long‐term study on crop pests and pathogens is required to account for factors beyond cropland management, such as climate change (Cilas et al. 2016). Altitude could also be a factor to consider when planning for the management of pests and pathogens. For example, cooler temperatures or better‐drained soils decrease Fusarium chlamydospore germination (Orr and Nelson 2018). CBB infestation can also be decreased at higher altitudes (Wegbe et al. 2003; Asfaw et al. 2019). Indigenous communities in the area tend to invest more time in growing crops that had more market and higher selling values the year before; thus, pests and diseases can fluctuate between years. It is also important to test specific interactions between pests and pathogens and other crops/trees to better understand mechanisms of competitive exclusion. Understanding pest and pathogen variability and resistance to insecticides and fungicides is also essential given their genetic plasticity (Ojua and Abu 2025). At the moment, the use of fungicides to limit fungal pathogens in the study area is not advised since we found no significant difference in fungal infestation between the community forest (where fungicides are forbidden by social rules) and the polyculture (with no limitation on the use of fungicides). Studies on the natural resistance of varieties of crops should also be incentivised. For example, different cocoa varieties have different resistance to black pod disease (Ofori et al. 2023), which is the main issue in the area. The removal of affected branches with a timely intervention is also very important in limiting the black pod disease in addition to improving cropland diversity (Adeniran et al. 2024); although this might not be sufficient in limiting some species of *Phytophthora* that are more aggressive (Ndoumbe‐Nkeng et al. 2004). The potential to use alternatives to chemicals such as dolomite and ash (currently used by some farmers) needs to be assessed in detail.

## 5 Conclusion

This study highlights the complex relationship between land management, biodiversity and pest and pathogen incidence in agroforestry systems. Our findings reinforce that while biodiversity can enhance natural pest and pathogen control and potentially improve yields for some crops, crop‐specific management strategies should be adopted. More needs to be done to understand crop‐specific mechanisms linked with competitive exclusion and natural control of pests and pathogens. Also, climate change can increase pest and pathogen incidence, so it is important to understand which varieties and crops are more resistant, potentially relying more on Indigenous crops (e.g., nutmeg and konjac) that are more resistant to pests and pathogens. But what is evident from the Indigenous communities of investigation is that their approach is to have a diversity of profits from different crops, thus buffering the negative effects of market fluctuations and yield loss. In Bali, management strategies for farming practices need to consider the Hindu cosmology concept of ‘Tri Hita Karana’ that emphasises the importance of relationships between humanity, religion, animals and the environment (Olthoff et al. 2026). Ultimately, ensuring the long‐term resilience of tropical agriculture requires a holistic approach that accounts for biodiversity, environmental variability, and Indigenous farming practices and culture.

## Author Contributions

**Marco Campera:** conceptualization (lead), data curation (equal), formal analysis (lead), funding acquisition (lead), methodology (lead), project administration (lead), resources (lead), software (lead), supervision (lead), validation (lead), visualization (lead), writing – original draft (lead), writing – review and editing (lead). **Jake Skull:** data curation (equal), investigation (equal), writing – original draft (supporting). **Lizzie H. Morton:** data curation (equal), investigation (equal), writing – original draft (supporting). **Charlotte A. Grant:** data curation (equal), investigation (equal), writing – review and editing (supporting). **Diogo De Almeida Santos:** data curation (equal), investigation (equal), writing – review and editing (supporting). **Dylan Wimble:** data curation (equal), investigation (equal), writing – review and editing (supporting). **Aislinn Olthoff:** data curation (equal), investigation (equal), writing – review and editing (supporting). **Lilli Stenger:** data curation (equal), investigation (equal), writing – review and editing (supporting). **Joel Bowden‐Pickstock:** data curation (equal), investigation (equal), writing – review and editing (supporting). **I. Made Setiawan:** conceptualization (supporting), project administration (supporting), resources (supporting), writing – review and editing (supporting). **I. Ketut Maliawan:** project administration (supporting), resources (supporting), writing – review and editing (supporting). **Angel Sangha:** data curation (equal), investigation (equal), writing – review and editing (supporting). **Jessica Chavez:** funding acquisition (supporting), investigation (equal), writing – review and editing (supporting). **Mia Garza:** data curation (equal), investigation (equal), writing – review and editing (supporting). **Jenna Sleath‐Probets:** data curation (equal), investigation (equal), writing – review and editing (equal). **Aikaterina Karageorgiadi:** data curation (equal), investigation (equal), writing – review and editing (supporting). **Made Dwi Sadnyana:** conceptualization (supporting), investigation (equal), methodology (supporting), project administration (supporting), resources (supporting), writing – review and editing (supporting). **I. Putu Leo Ardhiyanto:** investigation (equal), writing – review and editing (supporting). **Michela Balestri:** data curation (equal), writing – review and editing (supporting). **Andrew K. Jones:** supervision (supporting), writing – review and editing (supporting). **Sophie Manson:** investigation (equal), writing – review and editing (supporting). **Muhammad Syirazi:** resources (supporting), writing – review and editing (supporting). **Zefanya Ajiningrat Wibowo:** data curation (equal), project administration (supporting), resources (supporting), writing – review and editing (supporting). project administration (supporting), resources (supporting), supervision (supporting), writing – review and editing (supporting). **Vincent Nijman:** project administration (supporting), resources (supporting), supervision (supporting), writing – review and editing (supporting). **Matthew W. Bulbert:** project administration (supporting), supervision (supporting), writing – review and editing (supporting). **Vinni Jain:** data curation (equal), investigation (equal), methodology (supporting), project administration (supporting), supervision (supporting), writing – review and editing (supporting). **Desak Ketut Tristiana Sukmadewi:** data curation (supporting), project administration (supporting), resources (supporting), supervision (supporting), writing – review and editing (supporting).

## Funding

This work was supported by the Royal Geographical Society, Fieldwork Grant. Oxford Brookes University, HLS Developing Potential Research Excellence Award, Rolling Impact Development Award and Nigel Groome Studentship. People, Plants, Primates Action Fund, NA, Turing Sheme, NA, Oxford Brookes University Nigel Groome Studentship, Oxford Brookes University Rolling Impact Development Award.

## Conflicts of Interest

The authors declare no conflicts of interest.

## Data Availability

The data that support the findings of this study are openly available in RADAR at https://radar.brookes.ac.uk/radar/items/ba6b7134‐80d7‐43b3‐a7c5‐7e921b39adfd/1/.
